# ADMA: A Key Player in the Relationship between Vascular Dysfunction and Inflammation in Atherosclerosis

**DOI:** 10.3390/jcm9093026

**Published:** 2020-09-20

**Authors:** Laura Dowsett, Erin Higgins, Sarah Alanazi, Noha A. Alshuwayer, Fiona C. Leiper, James Leiper

**Affiliations:** 1 Institute of Cardiovascular and Medical Sciences, College of Medicine, Veterinary, and Life Sciences, The University of Glasgow, Glasgow G12 8QQ, UK; laura.dowsett@glasgow.ac.uk (L.D.); e.higgins.1@research.gla.ac.uk (E.H.); 2028273A@student.gla.ac.uk (S.A.); 2375058A@student.gla.ac.uk (N.A.A.); fiona.leiper@glasgow.ac.uk (F.C.L.); 2 Department of Pharmacology and Toxicology, College of Pharmacy, Al-Jouf University, Sakaka P.O. Box 2014, Saudi Arabia; 3 Department of Anatomy, College of Medicine, King Saud University, Riyadh 11451, Saudi Arabia

**Keywords:** asymmetric dimethylarginine, atherosclerosis, dimethylarginine dimethylaminohydrolase, endothelial dysfunction, plaque formation

## Abstract

Atherosclerosis is a chronic cardiovascular disease which increases risk of major cardiovascular events including myocardial infarction and stroke. Elevated plasma concentrations of asymmetric dimethylarginine (ADMA) have long been recognised as a hallmark of cardiovascular disease and are associated with cardiovascular risk factors including hypertension, obesity and hypertriglyceridemia. In this review, we discuss the clinical literature that link ADMA concentrations to increased risk of the development of atherosclerosis. The formation of atherosclerotic lesions relies on the interplay between vascular dysfunction, leading to endothelial activation and the accumulation of inflammatory cells, particularly macrophages, within the vessel wall. Here, we review the mechanisms through which elevated ADMA contributes to endothelial dysfunction, activation and reactive oxygen species (ROS) production; how ADMA may affect vascular smooth muscle phenotype; and finally whether ADMA plays a regulatory role in the inflammatory processes occurring within the vessel wall.

## 1. Introduction and Overview

In 1992, Vallance et al. were the first to observe that asymmetric dimethylarginine (ADMA) was elevated in chronic kidney disease patients [1]. Nearly 30 years on, raised ADMA is recognised as a hallmark of endothelial dysfunction [2], and, as such, related to many cardiovascular risk factors hypertension [3], obesity [4,5], hypertriglyceridemia [6] and type 1 [7] and type 2 diabetes [8]. In addition, longitudinal studies reveal that ADMA predicts cardiovascular morbidity and mortality in both myocardial infarction (MI) [9] and stroke patients [10]. Crucially, these studies have demonstrated that ADMA-driven cardiovascular risk is conserved, even when traditional and non-traditional risk factors are controlled. This review intends to explore the association between plasma ADMA and atherosclerosis, and to identify the potential causal mechanisms underlying ADMA’s role in the vascular and inflammatory processes involved in plaque formation.

The methylation of arginine residues is one of a myriad of post-translational modifications. ADMA, along with symmetric dimethylarginine (SDMA) and NG-mono-methylated-L-arginine (L-NMMA), is produced by protein arginine methyltransferase (PRMT) enzymes [11]. Nine PRMT enzymes have been identified to date, with all of them capable of forming mono-methylated-L-arginine. Type 1 PRMTs, of which PRMT1 is the most promiscuous, include PRMTs 3, 4 (also known as CARM1), 6 and 8, which catalyse the production of ADMA. In contrast, type 2 PRMTs, of which PRMT5 is the best described, are responsible for the formation of SDMA. In all cases, however, there are currently no known demethylation steps for arginine and, as such, ‘free’ methylarginines are released into the cytosol following proteolysis and can be transported into the circulation by cationic amino acid transporters [12]. ADMA and L-NMMA have been of interest to vascular scientists since they were shown to be endogenous inhibitors of nitric oxide synthase (NOS) via competition with L-arginine, the requisite NOS substrate and structural analogue of ADMA and L-NMMA [1,13]. In healthy serum, the concentration of ADMA is between 0.4 and 0.6 µM [14]; in contrast, the serum concentration of L-NMMA is 5–10 fold lower at ~0.05 µM [15]. The third methylarginine SDMA is found at concentrations similar to that of ADMA [14]. Although not discussed further in this review, SDMA has also been identified as an independent cardiovascular risk factor and predictor of all-cause mortality. Albeit that SDMA is not a direct inhibitor of NOS, SDMA can still alter L-arginine concentrations via competition at amino acid transporters. However, the biological relevance of this and further unknown mechanisms still needs to be investigated to fully understand how SDMA affects clinical outcome [15].

Arginine methylation is a constitutive process and, as such, free ADMA concentrations would quickly become elevated if not regulated, with resultant physiological consequences. In human volunteers, a pathophysiological bolus dose of ADMA significantly increased blood pressure [16] and inhibited forearm flow-mediated dilatation [17], consistent with inhibition of NOS. Renal excretion accounts for approximately 20% of the elimination of endogenous ADMA, with plasma ADMA elevated in renal failure patients [1,18,19]. The primary route of elimination involves the metabolism of ADMA by dimethylarginine dimethylaminohydrolase 1 and 2 (DDAH1 and DDAH2) to citrulline and dimethylamine. Given the importance of DDAH in ADMA clearance, several in vivo studies have been undertaken to understand whether DDAH dysfunction plays a causal role in cardiovascular disease.

Work by our group and others has shown that both heterozygous and homozygous DDAH1 deficiency, via the disruption of either exon 1 or 4, increased serum ADMA and ADMA accumulation in tissues. These findings were associated with a broad derangement of cardiovascular function, including elevated blood pressure, increased systemic vascular resistance and impaired vasoreactivity [20,21,22]. Conversely, overexpression of DDAH1 via viral transfection and genetic strategies lead to reduced ADMA levels with a corresponding decrease in blood pressure [23,24]. Interestingly, global deletion of DDAH2, through targeted disruption of exon 2, did not alter plasma ADMA but did lead to increased urinary, myocardial and renal ADMA accumulation with a resultant fall in plasma total nitrate and nitrite (NOx) concentrations [25]. This was associated with decreased endothelial-dependent relaxation and increased vasoconstriction, with systemic blood pressure raised during periods of activity. Overexpression of DDAH2 decreased accumulation of ADMA but had no influence on blood pressure [26]. Subsequent examination of DDAH2^-/-^ mice has revealed that ADMA accumulation occurs in the retina following oxygen-induced retinopathy and that this was associated with improved revascularisation [27]. Therefore, evidence suggests that while ADMA can promote vascular dysfunction in many settings, ADMA may also mediate protective effects in the vasculature due to its role in inflammatory cells. Finally, a third enzyme, alanine-glyoxylate aminotransferase-2 (AGXT2), accounts for a limited amount of ADMA metabolism. As with DDAH, we found AGXT2 deletion led to elevated ADMA and higher blood pressure [28]. Together, these in vivo observations support the notion that altered ADMA concentrations and metabolism have a causal role in the development of vascular derangement.

## 2. Clinical Association of ADMA with Atherosclerosis

The association between elevated plasma ADMA and the traditional risk factors for atherosclerosis has led to numerous clinical studies to determine whether ADMA is an independent factor in the development of atherosclerosis itself. Hypercholesteremia is a known driver of plaque formation, providing the low-density lipoprotein (LDL) cholesterol that forms the basis of the fatty lesion [29]. ADMA concentrations have been correlated with plasma LDL [30], and a lower L-arginine/ADMA ratio detected in hypercholesteraemic subjects [31].

Carotid artery intima–media thickness (IMT) is often used as a marker of atherosclerotic development in otherwise healthy individuals. Multiple studies have demonstrated a strong relation between increased IMT and elevated ADMA independent of other risk factors in healthy volunteers [32,33]. Flow-mediated dilatation (FMD) of the brachial artery, a common site of atherosclerosis, is also strongly associated with carotid artery IMT [34]. In a cohort of aged participants, FMD was negatively associated with the L-arginine/ADMA ratio. However, no association was observed with ADMA [35]. In contrast, analysis of the Young Finns study found that elevated plasma ADMA concentrations were associated with reduced FMD. Although male sex was a significant predictor of ADMA, there was no significant sex difference on the association between ADMA and FMD [36].

Interestingly, a number of prospective studies have also demonstrated that high ADMA is predictive of coronary artery disease (CAD). Furuki et al. determined that there was a significant relationship between baseline ADMA concentrations and IMT after a 6 year follow up in a healthy Japanese population [37]. There are suggestions that ADMA may exhibit site-specific promotion of subclinical atherosclerosis, as demonstrated by the Framingham Offspring Cohort Study which found ADMA concentrations were not linked with common carotid artery thickness but were found to affect the carotid bulb and internal carotid artery [38]. The Shimane CoHRE cross-sectional study, however, found no association between IMT with either ADMA or L-arginine concentration alone, whilst a decreased L-arginine/ADMA ratio did associate with IMT, thus suggesting that the L-arginine/ADMA ratio may be a more sensitive marker for atherosclerosis [39].

The importance of the ADMA-metabolizing enzymes, DDAH and AGXT2, has also been confirmed in clinical studies of CAD. Plasma ADMA levels are influenced by genetic variation in both DDAH1 [40,41,42] and DDAH2 [40,43]. In Chinese populations, a DDAH1 polymorphism, the promotor variant -396 4N, confers an increased risk of stroke, thrombosis and CAD [41]. Smaller cohort studies which have demonstrated a positive association between ADMA accumulation and DDAH1 genotype have been linked to hypertension [44] and CAD [42]. Interestingly, recent work in CAD patients has indicated that CAD incidence and ADMA levels are increased by AGXT2 variants rs37369 and rs16899974 [42].

In contrast to the above studies, Zsuga et al. found a negative relationship between ADMA and IMT, suggesting a protective effect of ADMA in inflammatory conditions characterized by increased iNOS [45]. They suggest that the extent of ADMA elevation is key in determining whether iNOS or eNOS blockade occurs in the disease process.

Meta-analyses agree there is substantial variation in the effect of ADMA. However, Xuan et al. still concluded that overall ADMA concentrations are significantly increased in CAD patients [46]. Upon further analysis, this correlation remained in patients with either MI or angina pectoris but there was no such relationship in those with acute coronary syndrome or slow coronary flow. Bai et al. again concluded that high plasma ADMA levels were associated with carotid IMT, with this link being stronger in subjects with chronic kidney disease [47].

In patients with CAD, ADMA was found to be an independent predictor of mortality in patients admitted with MI [48] and to have prognostic value beyond traditional risk factors in predicting further cardiovascular events [49]. Similarly, the Population Study of Women in Gothenburg revealed that systemic ADMA levels predicted the likelihood of both myocardial infarction and stroke over a 24 year period [9]. Furthermore, a meta-analysis by Willeit et al. observed a positive correlation between ADMA and risk of stroke [50]. Together, these human studies show a strong association between elevated ADMA concentrations on the development of CAD and contribute to worse outcome with increased likelihood of MI. However, these human studies cannot determine whether ADMA has a causal role in the pathogenesis of atherosclerosis.

## 3. Underlying Mechanisms of Atherosclerosis

It is well understood that atherosclerosis is both a vascular and inflammatory disease and this has been reviewed extensively [51,52]. However, to outline where ADMA may play a mechanistic role, atherosclerotic lesion formation and development will be briefly described here. In normal physiology, shear stress caused by high laminar blood flow protects the endothelium by suppressing proinflammatory factors and enhancing the release of vasoactive mediators such as NO, which as well as regulating vascular tone have anti-proliferative and anti-thrombotic effects. Lesions, in contrast, tend to occur in areas subjected to low shear stress and disturbed flow such as arterial branch points and areas of high vessel curvature. These areas exhibit increased permeability, allowing macromolecules including oxidised LDL (oxLDL) to migrate into the subendothelial space [53]. The atherogenic modifications of migrated LDL particles mark them as proinflammatory, inducing inflammatory endothelial signalling pathways including nuclear factor kappa-light-chain-enhancer of activated B cells (NF-kB). Consequently, the expression of NF-kB-dependent genes are upregulated including adhesion molecules, cytokines and growth factors. Macrophages migrate into the vessel wall and actively ingest and accumulate oxLDL, converting to foam cells [29]. Smooth muscle cells proliferate, producing large quantities of extracellular matrix, forming a fibrous cap over what can become a necrotic core [54]. Stable lesions can limit blood flow, causing local ischemia. However, upon plaque rupture, a thrombotic state occurs, which can in turn trigger the catastrophic events of a MI or cerebral stroke.

Early animal models of atherosclerosis using a hypercholesterolemic diet documented high plasma levels of ADMA and endothelial dysfunction in both rabbits [55] and monkeys [56]. In addition, plasma ADMA is raised in genetic [57] and pharmacological murine models of atherosclerosis [26]. In wild-type C57BL/6J mice, exogenous ADMA treatment triggers the development of aortic lesions [58] and induces perivascular fibrosis and intimal thickening in the coronary microvasculature [59]. Treatment of proatherogenic apolipoprotein E-deficient (ApoE^-/-^) mice with exogenous ADMA also exacerbates aortic lesion burden [58]. To study the significance of DDAH1 in atherosclerosis pathogenesis, Jacobi et al. developed a DDAH1-overexpressing, ApoE^-/-^ mouse model. Compared to ApoE^-/-^ counterparts, ADMA was lowered in DDAH1-overexpressing mice, which resulted in improved endothelial relaxation, decreased aortic lesion burden, as well as reduced neointimal lesion area and CD68 macrophage staining in the brachiocephalic trunk [57]. Consistently, Hasegawa et al. showed that angiotensin-II (Ang-II)-induced intimal thickening and perivascular fibrosis of coronary microvasculature is prevented by DDAH2 overexpression [26]. Interestingly, DDAH1 overexpression in femoral artery injury models improves endothelial repair, suppresses neointima formation and reduces local CD45 leukocyte infiltration [60], suggesting that elevated plasma ADMA levels might impact on atherogenesis by inhibiting vascular repair processes. These findings implicate ADMA as a culprit molecule in the development of atherosclerosis.

### 3.1. Endothelial Activation

Endothelial activation is typified by reduced endothelial barrier function, enhanced expression of adhesion molecules and an altered secretory profile, which impacts inflammation, homeostasis, cell behaviour and vascular tone [61]. Given the association between ADMA and endothelial dysfunction, several studies have attempted to elucidate the mechanisms through which ADMA may affect atherosclerotic lesion formation.

The role of ADMA in endothelial barrier function has been explored by several studies, in vitro [62,63,64,65] and in vivo [62]. ADMA treatment of porcine pulmonary artery endothelial cells (ECs) increased FITC-dextran permeability and actin stress fiber formation, which could be normalised by overexpression of DDAH and attenuated by the protein kinase G (PKG) activator 8-Bromo-cGMP or adenoviral mediated ras-related C3 botulinum toxin substrate 1 (Rac1) overexpression. This effect was also evident in DDAH1^-/-^ pulmonary microvascular cells, where basal levels of permeability were increased. Importantly, the effect of DDAH1 deletion was conserved in vivo, where reduced pulmonary endothelial barrier function resulted in increased Evans Blue accumulation in the lung [62,66]. Wang et al. later showed that ADMA enhanced apelin-13 passage through an endothelial monolayer in a concentration- and time-dependent manner, leading to increased myosin light chain phosphorylation in cocultured vascular smooth muscle cells (vSMCs) [64]. ADMA treatment again led to actin re-organisation and a dispersal of adherens junctions which were ameliorated by both nicotinamide adenine dinucleotide phosphate (NADPH) oxidase inhibitor apocynin and the p38 mitogen activated protein kinase (MAPK) inhibitor SB203580, indicating that ADMA may influence EC permeability by mediating actin cytoskeleton organisation through reactive oxygen species (ROS) and p38 MAPK signalling. These findings suggest that an ADMA-mediated reduction in endothelial barrier integrity could contribute to oxLDL accumulation in atherosclerosis although this is still to be definitively investigated.

**Figure 1:**
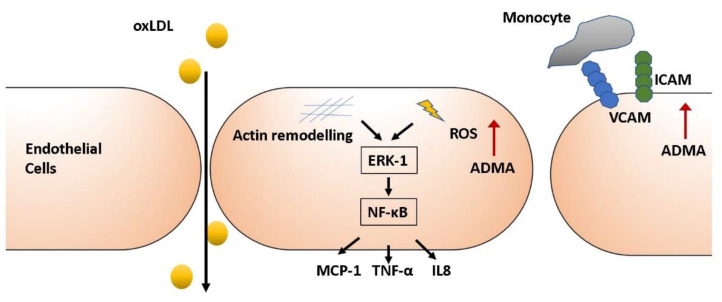
Asymmetric dimethylarginine increases endothelial activation. At the site of atherosclerotic lesions, inflammation leads to endothelial activation, reducing endothelial barrier integrity, enhancing the expression of adhesion molecules and increasing endothelial production of inflammatory markers. Asymmetric dimethylarginine (ADMA) treatment of endothelial cell monolayers increased endothelial permeability, increasing oxLDL transmigration. Actin cytoskeleton remodelling and reactive oxygen species (ROS) production, which are both induced by ADMA treatment, lead to the upregulation of ERK and NF-kB signalling and increased cytokine production. ADMA treatment also increases the expression of adhesion molecules ICAM and VCAM, leading to increased monocyte adhesiveness. Abbreviations: ADMA—asymmetric dimethylarginine, oxLDL—oxidised low-density lipoprotein, ROS—reactive oxygen species, ERK—extracellular signal related kinase, NF-kB—nuclear factor kappa-light-chain enhancer of activated B cells, ICAM—intracellular adhesion molecule, VCAM—vascular cell adhesion protein, MCP-1—monocyte chemoattractant protein 1, TNF α—tumour necrosis factor alpha, IL8—interleukin 8.

Under resting conditions, endothelial cell adhesion molecule expression and chemokine secretion is largely inhibited, keeping endothelial and circulating immune cell interaction to a minimum. Following inflammatory stimulation, ECs transition to an activated state, where they secrete a range of proinflammatory and chemotactic molecules, particularly monocyte chemoattractant protein-1 (MCP-1), Interleukin 6 (IL-6) and 8 (IL-8) [52]. Expression of P- and E-selectins and Ig-superfamily molecules intracellular adhesion molecule 1 (ICAM1) and vascular cell adhesion protein 1 (VCAM1), which mediate endothelial-leukocyte interactions and leukocyte transmigration into the subintimal space, are enhanced [67]. In human vascular disease, elevated ADMA is correlated with increased circulating levels of ICAM-1 [68] and VCAM-1 [69] and has been linked with increased circulating C-reactive protein (CRP), a prominent marker of EC activation [68,70]. These human studies have led to speculation that ADMA may influence EC activation and hence mediate proinflammatory actions in the endothelium (Figure 1). In vitro, ADMA treatment induced endothelial expression of ICAM-1 [63,71], VCAM-1 [72], the oxLDL receptor LOX-1 [73] as well as the proinflammatory cytokines MCP-1 [74] and tumour necrosis factor (TNF)-α [75]. Chan et al. demonstrated a functional effect where not only did ADMA dose-dependently enhance monocytoid cell adhesion to ECs in vitro but that plasma the L-arginine/ADMA ratio correlates inversely with peripheral blood mononuclear cell adhesion in hypercholestremic patients [30].

NF-κB is a key transcription factor in proinflammatory endothelial activation. Targeted endothelial inhibition of NF-κB signalling in ApoE^-/-^ mice reduced macrophage recruitment and decreased lesion area [76]. Consistently, ADMA has been shown to activate NF-κB in ECs [71,72,74,75]. Guo et al. demonstrated that ADMA increased nuclear translocation of NF-κB, which coincided with increased ICAM-1 expression in HUVECs. This was again associated with actin stress fiber formation and abolished following actin modulation by jasplakinolide and cytochalasin D [71]. Subsequent work by the group revealed that ADMA increased the degradation of NF-κB’s inhibitory partner, nuclear factor of kappa-light-chain polypeptide gene enhancer of B- cells inhibitor (IκBα), and facilitated NF-κB p65 subunit/actin interaction. Actin fiber disruption had no effect on ADMA-mediated IκBα degradation, but both NF-κB translocation and NF-κB p65/actin association were impaired, ameliorating ADMA-mediated NF-κB activation and VCAM-1 expression [72]. These observations would suggest that the proinflammatory actions of ADMA in the endothelium are dependent on actin cytoskeleton modulation. Other studies report that ADMA-induced NF-κB activation and soluble ICAM-1 expression is concomitantly linked with p38 MAPK and extracellular signal related kinases (ERK) 1/2 phosphorylation [75]. Interestingly, inhibition of p38 MAPK and ERK1/2 attenuated the effect of ADMA on both ICAM expression and NF-κB activation. Accordingly, ADMA-induced NF-κB activation appears to be regulated upstream by p38 MAPK and ERK1/2 signalling. Given the central role of NF-κB, its modulation by ADMA could be a significant mechanistic link through which ADMA impacts the endothelial inflammatory state. It will be interesting to determine whether ADMA affects other NF-κB downstream targets such as cyclooxygenase-2 and matrix metalloproteinase expression and assess their role in endothelial activation.

Scalera et al. demonstrated that ADMA treatment of human umbilical vein endothelial cells (HUVECs) reduced telomerase activity, enhanced telomere attrition and decreased β-galactosidase activation, consistent with cellular aging and senescence [77]. In addition, ADMA-induced senescence was linked with MCP-1 and IL-8 expression and was sensitive to pyrrolidine dithiocarbamate antioxidant treatment. Given that NF-κB is an established mediator of ROS-dependent MCP-1 and IL-8 expression, Scalera et al. postulated that ADMA-induced senescence is ROS dependent, and that again NF-κB signalling may be implicated. EC senescence is enhanced in atherosclerotic lesions and is associated with a reduction in eNOS activity in vitro [78]. Taken together, these data suggest that ADMA may mediate atherogenesis through reduced endothelial barrier function, altered NF-κB signalling and actin fiber re-organisation, accelerating EC senescence and promoting endothelial inflammation and dysfunction.

### 3.2. Oxidative Stress in Endothelial Cells

As briefly mentioned above, the overproduction of reactive oxygen species (ROS) is a feature of many cardiovascular pathologies. In the coronary vasculature, enhanced oxidative burden is an important hallmark of atherosclerosis progression and lesion instability [79], with systemic levels of ROS predicting cardiovascular events in CAD [80]. ADMA accumulation, endothelial dysfunction and oxidative stress have been independently linked to CAD [81]. ADMA and ROS both disrupt NO signalling, suggesting that they may interact to cause, or reinforce, vascular impairment and disease [81,82]. Antoniades et al. demonstrated that high serum ADMA correlates with enhanced superoxide production in both saphenous veins and internal mammary arteries derived from CAD patients [83]. However, to date, the association between ADMA and vascular ROS levels in CAD has not been extensively examined in a clinical setting.

The protective effects of vascular NO can, in part, be attributed to ROS scavenging and activation of endogenous antioxidant defense systems which act to mitigate effects of vascular ROS accumulation [84]. Thus, by reducing NO bioavailability, ADMA may promote vascular ROS imbalance contributing to endothelial dysfunction. However, ADMA is also thought to invoke superoxide production via eNOS uncoupling. Treatment of HUVECs with 1 mM ADMA increased superoxide formation by ~130% [83], while 10 μM ADMA caused a ~2-fold increase in production [74]. Böger et al. reported that ADMA-driven endothelial superoxide generation was accompanied by NF-κB activation and secretion of the chemotactic protein MCP-1 [74]. Suda et al. have shown that long-term ADMA treatment stimulated cardiac superoxide production, which was related to increased coronary artery lesion formation in mice [59]. Interestingly, lesion formation in eNOS-deficient mice was comparable to wild types, suggesting that ADMA can modulate oxidative burden via NO-independent mechanisms. ADMA may also inhibit antioxidant defence systems. In rats, chronic ADMA administration reduces systemic activity of superoxide dismutase (SOD), glutathione peroxidiase and catalase. Crucially, this was accompanied by increased circulating levels of the lipid peroxidation and oxidative stress marker malondialdehyde [85]. Accordingly, ADMA not only contributes to ROS burden through multiple mechanisms, but may also reduce ROS disposal, therein establishing a pathogenic redox state in the vasculature. ADMA signalling itself may also be regulated by ROS; in rat gracilis muscle arterioles, ADMA concentration-dependent contraction was abolished by the presence of SOD [86]. Veresh et al. later demonstrated that the effect of ADMA on basal tone and flow-mediated dilatation was prevented by both endothelial denudation and the NADPH inhibitor apocynin, implicating superoxide generation and endothelial function as requisite mediators in the vascular response to ADMA [87]. Together, these findings indicate a causal role for ADMA in establishing oxidative stress driven vascular damage. However, whether ADMA can invoke ROS-mediated endothelial dysfunction at pathologically relevant concentrations seen in atherosclerosis is still unclear.

Of note, while ADMA potentially acts to induce vascular oxidation, studies in vitro show that DDAH activity may be reduced under conditions of nitrosative [88] and oxidative stress [89]. Furthermore, oxLDL caused a reduction in endothelial DDAH1 activity, which coincided with ADMA formation [90]. Conversely, PRMT expression and ADMA levels in ECs are positively regulated by oxLDL [91]. This would suggest that ADMA levels are likely to be increased in response to enhanced ROS signalling. In the context of endothelial dysfunction, positive feedback loops involving ROS elaboration and its reciprocal interactions with proatherogenic factors such as oxLDL play a role in sustaining EC activation and amplifying atherosclerosis advancement [92]. It could be speculated that ROS regulation of enzymatic mediators of ADMA metabolism, in concert with ADMA-driven ROS production, may also be an important pathogenic mechanism in vascular disease.

### 3.3. Plaque Formation

The proliferation of smooth muscle cells drives the formation of a fibrous cap under which inflammatory cells accumulate and eventually form a necrotic core [54]. Lesions with a large fibrous cap are thought to be stable and, although these can cause local ischemia, are protective against thrombotic events. In contrast, a thin smooth muscle layer promotes instability, with rupture triggering a thrombotic cascade, which can lead to major cardiovascular events including MI and stroke. Most clinical studies associating ADMA plasma concentrations with atherosclerotic development use markers of neointima hyperplasia or vessel calcification [32,33,37,38]. However, although EC-produced NO is a key regulator of vSMC function, there have been relatively few studies to determine whether ADMA has mechanism effects in smooth muscle proliferation, extracellular matrix deposition or plaque calcification.

Interestingly, although NOS is not expressed in vSMCs under basal conditions, DDAH is expressed constitutively. DDAH1 expression has been demonstrated by gene expression and Western blotting [93,94], while DDAH2 has been identified in both myofibrils and in smooth muscle nuclei [95]. This suggests that DDAH may be an important regulator of vSMC iNOS activity or be important for the metabolism of ADMA produced elsewhere. Endothelial-produced ADMA has been shown to act as a soluble signalling molecule on vSMCs, increasing their proliferation through the induction of ERK signalling [96]. Furthermore, in a model of pulmonary arterial hypertension, ADMA treatment has been shown to upregulate vSMC proliferation, likely through an upregulation of Rho kinase signalling [97]. Rho A and Rho kinase (ROCK) signalling has again been implicated in the ADMA-mediated increase in vSMC migration. ADMA treatment led to the increased phosphorylation of ROCK downstream target MYPT-1, which led to the reorganisation of the actin cytoskeleton and altered vSMC morphology. Treatment with the ROCK inhibitor Y27632 and overexpression of DDAH2 both reverse the ADMA-driven increase in vSMC migration [98].

The proinflammatory cytokines chemokine (c-c motif) ligand 5 (CCL5) and IL-1β are both thought to contribute to neointimal hyperplasia. Surprisingly, however, treatment of vSMCs with either cytokine increased DDAH1 expression, leading to suppression of ADMA accumulation and amplification of iNOS signalling [93,94]. Neither of these studies look beyond the effect on DDAH to determine how reducing ADMA effected vSMC proliferation. The conflicting evidence on the effect of ADMA on vSMC proliferation and migration suggests that further work in the setting of atherosclerosis is needed to determine how the interaction between ADMA signalling and inflammation regulates vSMC phenotype.

Stenting is a common treatment for CAD and, following stent placement, plasma ADMA concentrations fall, with a corresponding increase in arginine concentration, leading to a significant improvement in the arginine/ADMA ratio. This suggests that either the plaque itself or the associated ischemic condition is a factor contributing to raised ADMA concentration [99]. However, following stenting, ADMA is a determining factor as to whether re-stenosis occurs, with ADMA concentrations correlating with in-stent calcification and neoatherosclerosis [100]. Stenting can cause endothelial damage. Therefore, high ADMA concentrations can block endothelial re-growth, leading to neointimal growth. Konishi et al. found that DDAH1 overexpression rescued this phenotype, resulting in lower levels of proliferation and inflammatory cell infiltration [60]. These studies suggest that ADMA concentrations are key to successful treatment of atheromas, and methods to lower ADMA would be beneficial to improved outcomes of angioplasty patients.

### 3.4. Inflammation

Atherosclerosis is accompanied by chronic, low-grade inflammation, where oxidised LDL particles in the vessel wall lead to the recruitment of inflammatory cells to the lesion [29]. As discussed above, the activation of NF-kB in the endothelium promotes the expression of selectins and adhesion markers, which in turn trigger the transmigration of immune cells [52,67]. Although inflammatory mechanisms of atherosclerosis have been extensively reviewed [51], here we discuss how ADMA modulates this immune response.

Following migration to the lesion, monocytes differentiate into the proinflammatory M1 macrophage. Classically activated M1 macrophages release a variety of proinflammatory cytokines, chemokines and reactive oxygen species which contribute to the maintenance of the local inflammatory response [51]. The expression of iNOS and NO production are also hallmarks of M1 macrophages, contributing to tissue damage; given the status of ADMA as a NO inhibitor, its involvement in macrophage iNOS signalling is of interest. Early studies by Macallister et al. demonstrated that ADMA inhibited NO production in cultured human and murine macrophages in a concentration-dependent manner [101]. Similarly, a more recent study demonstrated ADMA as a regulator of LPS-induced NO production in macrophages via inhibition of NF-kB activity and consequent suppression of iNOS mRNA and protein expression [102]. ADMA also enhanced the formation of superoxide in macrophages in vivo, identifying a negative correlation between an ADMA-dependent decrease in NO production and an ADMA-dependent increase in superoxide release [102]. In inflammatory cells, ADMA metabolism is confined to DDAH2 and, we have, therefore, investigated the impact of DDAH2 deficiency on macrophage function [25,103]. As expected, ADMA concentration was notably higher in DDAH2^-/-^ peritoneal macrophages, with a corresponding reduction in NOx production following treatment with a polyfactorial proinflammatory treatment consisting of interferon-γ, LPS and TNFα. We demonstrated impaired stimulated macrophage phagocytic activity and motility in cells from both global and monocyte-specific DDAH2^-/-^ mice. Such findings indicate the ability of ADMA and impaired ADMA metabolism to negatively influence the balance in the production of reactive mediators during inflammatory states. However, whether ADMA alters NO production in models of atherosclerosis has not been investigated.

**Figure 2:**
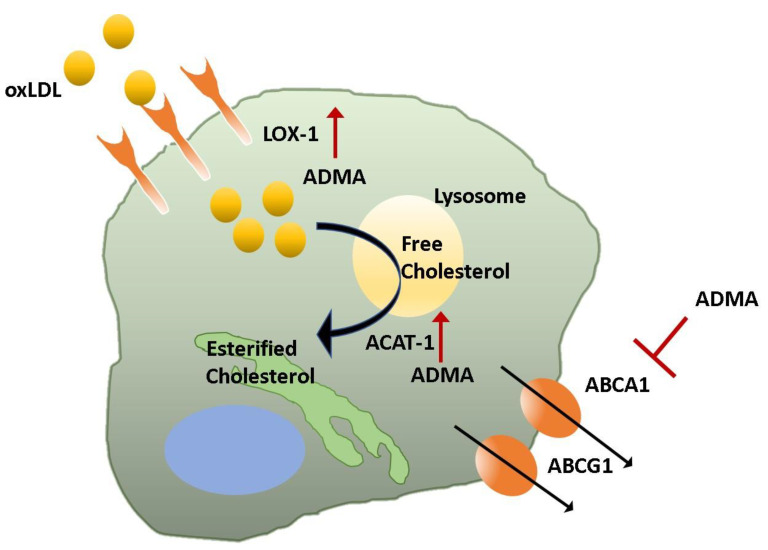
ADMA increases the generation of foam cells. Macrophages play a key role in scavenging oxidized lipids within the vessel wall; however, in atherosclerotic plaques, excessive lipid accumulation leads to the formation of foam cells. Macrophages express scavenger receptors, including lectin-like, oxidized, lipoprotein receptor-1 (LOX-1), which mediate the uptake of oxidized LDL (oxLDL). Once within the macrophage, oxLDL is taken up by the lysosome and converted to free cholesterol. Acetyl coenzyme A: cholesterol aceyltransferase-1 (ACAT) esterifies the cholesterol to be stored within the endoplasmic reticulum. Asymmetric dimethylarginine (ADMA) treatment of macrophages upregulates LOX-1 and ACAT-1, increasing cholesterol storage. ADMA also downregulates the expression of key ATP-binding cassette (ABC) transporters ABCA1 and ABCG1, thereby reducing cholesterol efflux from the macrophage and contributing to foam cell formation.

At the site of the lesion, differentiated macrophages express a high level of scavenger receptors including the lectin-like, oxidized, lipoprotein receptor (LOX-1). Scavenger receptors, including LOX1, play a crucial role in early atherogenesis and are involved in LDL uptake and subsequent generation of foam cells. Smirnova et al. found that treatment of PMA-primed human monocytic leukaemia cells with ADMA significantly increased the expression of LOX-1 by 217% (Figure 2). This increase was coupled with a more than 2-fold upregulation of oxLDL accumulation [104]. Following uptake, oxLDL is degraded by lysosomes, generating free cholesterol to be trafficked to the endoplasmic reticulum and esterified by Acyl-coenzyme A: cholesterol acyltransferase (ACAT). In THP-1-derived macrophages and foam cells, ADMA treatment increased the expression of ACAT-1, leading to increasing cellular cholesterol content in a time- and concentration-dependent manner [105]. Since LDL cholesterol upregulates the generation of ADMA [91] and ADMA increases oxLDL uptake, it would suggest a positive feedback loop, where ADMA concentrations drive foam cell formation within the atherosclerotic plaque.

The ATP-binding cassette (ABC) transporters ABCA1 and ABCG1 are regulated by the nuclear liver x receptor (LXR) which is activated by cholesterol metabolites. When macrophage function is impaired, their ability to effectively efflux cholesterol is reduced, contributing to foam cell formation. Interestingly, a recent study proposed that ADMA treatment of macrophages reduced liver X receptor alpha (LXRα) activity, downregulating the expression of ABCA1 and ABCG1 transporters via the activation of NADPH oxidase ROS generation. As a result, cholesterol efflux was impaired, and ADMA drove excessive macrophage lipid accumulation [106]. Overexpression of DDAH2 was protective and returned cholesterol efflux to normal levels. Failure of proper clearance of lipids can lead to macrophage apoptosis, contributing to the development of a necrotic core. ADMA treatment of THP-1 cells induced ER stress and increased caspase 4 activity, eventually leading to higher levels of apoptosis [107]. Together, these data suggest that while ADMA may suppress inflammatory processes via iNOS inhibition, ADMA also promotes foam cell formation, a key driver in plaque development. Therefore, the balance of these processes needs to be fully understood in vivo to determine whether the regulation of ADMA metabolism in innate immune cells is a viable target for the treatment of atherosclerosis.

While lipid-laden macrophages are the predominant immune cell type within an atherosclerotic plaque, there are also a significant number of T cells found within the lesion. It is now clear that the adaptive immune response plays a significant role in the development and progression of atherosclerosis. It has been suggested that differing subsets of T cells play opposing roles in atherogenesis although compensatory mechanisms have left much still to be determined [108]. Unlike the extensive literature relating to the impact of ADMA/NO signalling in macrophages, very little is known about this pathway in T cells and this is still a field of research for future study.

Recent advances in the treatment of CAD have focused on chronic inflammation playing an active role in the development of atherogenic disease. A number of trials, including COLCOT and CANTOS, have shown that intervening in IL-1β or IL6 signalling pathways is successful in improving patient outcome [109,110]. A number of clinical studies have shown a positive association between high concentrations of IL-6 and ADMA in patients with CAD and those undergoing angioplasty [111,112]; similar associations have also been demonstrated in rheumatoid arthritis [111] and septic shock [113]. However, as with all studies which show association, there is no mechanistic insight as to the relationship between interleukin signalling and ADMA concentration and it would be of future interest to understand whether ADMA/NO signalling was affected in the major inflammatory trials.

## 4. Conclusions

Although there have been major advances in the treatment of atherosclerotic plaques, their frequency within the population contributes to the high rates of catastrophic cardiovascular events including MI and stroke. Furthermore, although angioplasty and stenting have been a breakthrough in the management of lesions, they do not always maintain longevity due to neointima formation and restenosis. As a result, understanding the mechanisms which underly plaque formation is key to the development of new treatments. Elevated plasma ADMA not only correlates with the presence of plaques, particularly in the carotid artery, but also predicts risk of future lesion development, myocardial infarction and stroke. It is, therefore, important to determine the mechanisms underlying ADMA-related risk. In vivo and in vitro studies clearly demonstrate that exogenous ADMA and impaired ADMA metabolism have a profound effect on endothelial function, reducing barrier integrity while increasing inflammatory markers and ROS production. Many of these effects are through the inhibition of NO production, although the dependence on NO is not clear in all studies. The role of ADMA in inflammatory cells, however, is not as clear. Proatherogenic pathways via foam cell formation would again suggest that ADMA plays a key role in lesion formation. However, a number of studies have indicated that ADMA can be beneficial in controlling chronic inflammatory pathways via the suppression of iNOS signalling. Given that inflammatory cells are unique in only expressing DDAH2, this may make this an interesting target in controlling the inflammatory processes in established disease.
